# Exploring genetic diversity and phylogenic relationships of Chinese cattle using gene mtDNA 16S rRNA

**DOI:** 10.5194/aab-62-325-2019

**Published:** 2019-06-12

**Authors:** Linjun Yan, Yifan She, Mauricio A. Elzo, Chunlei Zhang, Xingtang Fang, Hong Chen

**Affiliations:** 1 Institute of Cellular and Molecular Biology, Jiangsu Normal University, Xuzhou, Jiangsu 221116, China; 2 School of Environmental and Biological Engineering, Nantong College of Science and Technology, Nantong, Jiangsu 226007, China; 3 Department of Animal Sciences, University of Florida, Gainesville, FL 32611-0910, USA

## Abstract

The objective of this research was to characterize the genetic diversity and phylogenetic diversity among 12 cattle breeds (10 Chinese breeds and two foreign taurine breeds as controls) utilizing gene mtDNA 16S rRNA. The complete sequences of the mtDNA 16S rRNA genes of the 251 animals were 1570 bp long. The mean percentages of the four nitrogen bases were 37.8 % for adenine (A), 23.7 % for thymine (T), 20.9 % for cytosine (C), and 17.6 % for guanine (G). The mtDNA 16S rRNA gene base percentages had a strong bias towards A + T. All detected nucleotide variations in gene mtDNA 16S rRNA were either transitions (62.3 %) or transversions (37.7 %); no indels (insertions and deletions) were found. A total of 40 haplotypes were constructed based on these mutations. A total of 36 haplotypes of these 40 haplotypes were present in 10 Chinese cattle breeds. The haplotype diversity of all Chinese cattle populations was 0.903±0.077, while the nucleotide diversity was 0.0071±0.0039. Kimura's two-parameter genetic distances between pairs of the studied 12 breeds ranged from 0.001 to 0.010. The phylogenetic analysis assigned the 10 Chinese breeds to two distinct lineages that likely differed in their percentage of *Bos taurus* and *Bos indicus* ancestry.

## 1 Introduction

As some of the most economically important domestic animals in the world, cattle have been attracting close attention for a long time. To improve conservation and rational utilization, many scientists focus their research on the origin, phylogenetic relationships, and genetic diversity of this species. Mitochondrial DNA (mtDNA) has been widely used in genetic studies because of its maternal inheritance, absence of introns, the existence of single-copy orthologous genes, lack of recombination events, and high mutation rate. The bovine mtDNA is a double-stranded circular molecule consisting of a displacement loop (D-loop) region and 37 genes. Its complete sequence was published by Anderson et al. (1982). Of the 37 genes, 13 code for proteins (polypeptides), 22 code for transfer RNA (tRNA), and two code for the small (12S) and large (16S) subunits of ribosomal RNA (rRNA). The D-loop region (Loftus et al., 1994; Bradley et al., 1996; Mannen et al., 1998; Kikkawa et al., 2003; Lai et al., 2006; Lei et al., 2006; Xia et al., 2019) and the cytochrome b (*cyt b*) gene (Lau et al., 1998; Sultana et al., 2003; Cai et al., 2003; Souza et al., 2009; Stock et al., 2009; Yap et al., 2010) of mtDNA have been widely used in cattle to characterize genetic diversity and phylogenetic performance among individuals and populations. The mtDNA 16S rRNA gene is also an advanced genetic marker for animal genetic diversity and phylogenetic studies. Most gene mtDNA 16S rRNA studies were with aquatic organisms (Canapa et al., 2000; Stillman and Reeb, 2001; Masaoka and Kobayashi, 2005), insects (Kambhampati et al., 1996; Nguyen et al., 2014), and a few mammalian species (Kuznetsova et al., 2005); no reports involved cattle. Thus, the objective of this research was to characterize the genetic diversity and phylogenetic diversity among 12 cattle breeds (10 Chinese breeds and two control foreign taurine breeds) utilizing gene mtDNA 16S rRNA. Polymorphic sites, nucleotide variation, and haplotype diversity were determined using complete sequences of the mtDNA 16S rRNA gene across the 12 cattle breeds. Further, a phylogenetic analysis based on genetic distances was carried out for the first time to explore relationships among the 12 cattle breeds.

## 2 Materials and methods

### 2.1 Ethics statement

The Institutional Animal Care and Use Committee (IACUC) of the School of Life Science of Jiangsu Normal University approved the animal study proposal, with the permit number SYXK(Su) IACUC 2011-0039. All cattle experimental procedures were performed in accordance with the Regulations for the Administration of Affairs Concerning Experimental Animals approved by the State Council of the People's Republic of China.

### 2.2 Sampling and DNA extraction

Whole blood samples of 251 individuals were obtained from 12 cattle breeds: 10 Chinese breeds (six native breeds and four foreign–native crossbreeds) and two foreign breeds (controls) present in various areas in the central, northern, and northwestern regions of China (Table 1). The six native breeds were Qinchuan (QC; n=22), Nanyang (NY; n=21), Jianxian (JX; n=22), Zaosheng (ZS; n=17), Mongolian (MG; n=17), and Enshi (ES; n=21). The four foreign–native crossbreeds were Xianan (XN; n=21), Chinese Holstein (HS; n=21), Red Steppe (HN; n=22), and Denan (DN; n=27). The two foreign breeds were Angus (AN; n=19) and Japanese Black (JB; n=21).

**Table 1:** Information on the 12 cattle breeds in the study.

Breed	Description	Acronym	Sample size	Geographical location
Qinchuan	Chinese native	QC	22	Central region, Shaanxi
Nanyang	Chinese native	NY	21	Central region, Henan
Jianxian	Chinese native	JX	22	Central region, Henan
Zaosheng	Chinese native	ZS	17	Central region, Gansu
Mongolian	Chinese native	MG	17	Northern region, Inner Mongolia
Enshi	Chinese native	ES	21	Southern region, Hubei
Xianan	Crossbreed (Charolais × Nanyang)	XN	21	Central region, Henan
Chinese Holstein	Crossbreed (Holstein × Chinesenative)	HS	21	Northwestern region, Shaanxi
Red Steppe	Crossbreed (Shorthorn × Mongolian)	HN	22	Northwestern region, Jilin
Denan	Crossbreed (Gelbvieh × Nanyang)	DN	27	Central region, Henan
Angus	Taurine Scottish breed (control)	AN	19	Northwestern region, Shaanxi
Japanese Black	Taurine Japanese breed (control)	JB	21	Northern region, Anhui
Total			251	

Blood samples were stored at -80 ${}^{\circ }$C until processing. Genomic DNA was extracted using a standard phenol chloroform extracting method (Sambrook et al., 1989). The leucocyte was separated from the whole blood samples and split in a solution containing sodium dodecyl sulfate (SDS) and proteinase K. Protein precipitation was implemented with phenol and chloroform, and genomic DNA was precipitated with isopropanol and ethanol. The concentration of extracted DNA was measured by a NanoDrop 2000 spectrophotometer (Thermo Scientific, USA), and was diluted to use as template for amplification.

### 2.3 Amplification and sequencing

The mtDNA 16S rRNA gene was amplified using forward (5${}^{\prime }$-GCATCCAGTTTACACCTAGA-3${}^{\prime }$) and reverse (5${}^{\prime }$-GCTCTGCCACCTTAACTA-3${}^{\prime }$) primers designed based on the complete sequence of the *Bos taurus* mtDNA genome (GenBank accession number AY526085). Polymerase chain reaction (PCR) was performed in a total volume of 30 µL, containing 10 ng of genomic DNA, 3 µL of 10× buffer (${\mathrm{Mg}}^{2+}$ plus), 200 µM of dNTP (dATP, dTTP, dCTP, and dGTP), 1 µM of each primer, and 1.5 units of Taq DNA polymerase (MBI, Shanghai, China). The PCR cycling was carried out in a PTC-200 thermocycler (MJ Research Inc., USA) under the following conditions: 3 min at 94 ${}^{\circ }$C, 35 cycles of denaturing at 94 ${}^{\circ }$C for 45 s, annealing at 57 ${}^{\circ }$C for 1 min, extension at 72 ${}^{\circ }$C for 90 s, and a final extension at 72 ${}^{\circ }$C before cooling to 4 ${}^{\circ }$C for 10 min. The PCR products were checked by electrophoresis on 1 % agarose gels and were visualized under UV light after staining with ethidium bromide. The PCR products were purified using a Wizard PCR Preps DNA purification kit (Promega, USA) according to the manufacturer's instructions, and were sequenced in both directions at a commercial laboratory (Sangon Biotech, Shanghai, China) in an ABI 377 DNA sequencer (Applied Biosystems, Foster City, CA, USA).

### 2.4 Gene sequence data and phylogenetic analysis

The mtDNA 16S rRNA gene sequences were edited using the DNASTAR 7.0 package (DNASTAR, Madison, WI). The program Clustal X version 2.0 (Larkin et al., 2007) was used for multiple sequence alignments. Numbers of nucleotide polymorphic sites, nucleotide diversity (π), haplotype diversity (h), and Tajima's D test were obtained with software DnaSP version 5.1 (Librado and Rozas, 2009). Nucleotide diversity (π) is the average number of differences between random pairs of homologous nucleotide sites in a sample (Nei and Li, 1979). Haplotype diversity (h) is the average number of differences between random pairs of homologous haplotype sequences in a sample (Nei, 1987). Tajima's D test compares the number of differences between pairs of haplotypes with the number of segregating nucleotide sites in a sample (Tajima, 1989).

Genetic distances based on a Kimura two-parameter model (Kimura, 1980) were computed with MEGA5.0 software (Tamura et al., 2011). A dendrogram was constructed according to the unweighted pair group method with arithmetic mean (UPGMA) method (Sokal and Michener, 1958). To find phylogenetic clusters, the software MEGA5.0 was utilized to construct a maximum likelihood (ML) tree using the Hasegawa–Kishino–Yano model (Hasegawa et al., 1985) with the following parameters: 1000 bootstrapping replicates, a gamma distribution (+G) with eight rate categories, and evolutionary invariability (+I). The ML trees only displayed reliability percentages above 30 %, and values above 50 % were considered highly reliable. A median-joining (MJ) network was constructed using the software NETWORK4.6.1.3 (Bandelt et al., 1999).

## 3 Results

### 3.1 Sequence composition and variation

The complete sequences of the mtDNA 16S rRNA genes of the 251 animals from the 12 cattle breeds were obtained. All sequences were 1570 bp long, and neither insertions nor deletions were found. The mean percentages of the four nitrogen bases were 37.8 % (37.4 % to 38.0 %) for adenine (A), 23.7 % (23.5 % to 23.9 %) for thymine (T), 20.9 % (20.6 % to 21.0 %) for cytosine (C), and 17.6 % (17.5 % to 18.0 %) for guanine (G). The mtDNA 16S rRNA gene base percentages had a strong bias towards A + T (61.5 %) in the 12 cattle breeds. No significant differences in nucleotide composition of the mtDNA 16S rRNA gene existed among the 10 Chinese and the two foreign cattle breeds.

A total of 78 polymorphic sites were identified, and 40 of them were parsimony-informative sites (sites with at least two types of nucleotides with a minimum frequency of two in two or more of them). The Qinchuan (QC) and Jiaxian (JX) breeds had more polymorphic sites (n=36) than any of the other cattle breeds, while the Japanese Black (JB; one of the controls) had the least number of polymorphic sites (n=12; Table 2). All detected nucleotide variations in gene mtDNA 16S rRNA were either transitions (62.3 %) or transversions (37.7 %).

**Table 2:** Genetic diversity indexes in 10 Chinese cattle breeds and two foreign breeds based on mtDNA 16S rRNA gene sequences.

Breeds	Polymorphic	Parsimony-	Number of	Haplotype	Nucleotide	Tajima's D	Statistical
(acronym)	sites	informative	haplotypes	diversity	diversity		significance
		sites		(h± SE)	(π± SE)		
Qinchuan (QC)	36	19	8	0.909 ± 0.065	0.0072 ± 0.0037	1.24758	P> 0.10
Nanyang (NY)	23	16	5	0.933 ± 0.029	0.0079 ± 0.0041	0.28781	P> 0.10
Jianxian (JX)	36	16	8	0.911 ± 0.078	0.0076 ± 0.0039	-0.22606	P> 0.10
Zaosheng (ZS)	25	22	2	0.965 ± 0.126	0.0095 ± 0.0049	1.16370	P> 0.10
Menggu (MG)	26	17	6	0.952 ± 0.096	0.0080 ± 0.0038	0.52178	P> 0.10
Enshi (ES)	30	4	4	0.818 ± 0.119	0.0042 ± 0.0029	-1.86325	0.01<P<0.05
Xianan (XN)	26	20	8	0.927 ± 0.066	0.0078 ± 0.0042	1.11318	P> 0.10
Chinese Holstein (HS)	34	24	9	0.969 ± 0.096	0.0102 ± 0.0053	-0.04635	P> 0.10
Red Steppe (HN)	17	10	5	0.811 ± 0.039	0.0040 ± 0.0031	-1.10475	P> 0.10
Denan (DN)	26	13	5	0.831 ± 0.051	0.0047 ± 0.0033	-1.60370	0.01<P<0.05
Angus (AN)	21	17	4	0.826 ± 0.045	0.0043 ± 0.0034	-0.67090	P> 0.10
Japanese Black (JB)	12	3	6	0.802 ± 0.096	0.0037 ± 0.0028	-1.18320	P> 0.10

### 3.2 Haplotype sharing and genetic diversity

A total of 40 haplotypes (Hap1 to Hap40) were identified by comparing the 251 individual animals' mtDNA 16S rRNA gene sequences from the 12 cattle breeds (Table 3). A total of 36 haplotypes of these 40 haplotypes were present in 10 Chinese cattle breeds, while the other four haplotypes were only found in the two foreign breeds. (i.e., Hap20 and Hap23 were only present in AN, while Hap25 and Hap40 were only present in JB). Hap4, present in 86 out of 251 samples (34.3 %), was found to be the dominant haplotype. Hap2, present in 21.9 % of the samples, ranked second, and Hap18, present in 11 samples (4.4 %), was a distant third. Most of the remaining haplotypes were represented in five or fewer samples.

**Table 3:** Distribution of haplotypes in 12 cattle breeds based on mtDNA 16S rRNA gene sequences.

	Breed${}^{*}$												
Haplotype	QC	NY	JX	ZS	MG	ES	XN	HS	HN	DN	AN	JB	Total
Hap1			1										1
Hap2	5	8	3	5		11	10	1		12			55
Hap3					2								2
Hap4	6	4	9	12	3		5	7	12	2	9	17	86
Hap5		5											5
Hap6	3				5	1							9
Hap7									4				4
Hap8								4					4
Hap9			3										3
Hap10	2												2
Hap11		2					2						4
Hap12							2						2
Hap13									2				2
Hap14	1		1										2
Hap15		1											1
Hap16	1		1					2					4
Hap17						2							2
Hap18										4			4
Hap19								1					1
Hap20											3		3
Hap21			2										2
Hap22									7				7
Hap23											1		1
Hap24	2												2
Hap25												2	2
Hap26			2										2
Hap27								2					2
Hap28					5						6		11
Hap29						2							2
Hap30					2								2
Hap31								1					1
Hap32	2												2
Hap33						5							5
Hap34							2						2
Hap35										4			4
Hap36								1					1
Hap37								2					2
Hap38									2				2
Hap39		1											1
Hap40												2	2
Total	22	21	22	17	17	21	21	21	27	22	19	21	251

${}^{*}$ Breed abbreviations given in Table 1.

The mean haplotype diversity of the 10 Chinese cattle breeds was 0.903±0.077, ranging from 0.811±0.039 (HN) to 0.969±0.096 (HS), whereas the mean nucleotide diversity was 0.0071±0.0039, ranging from 0.0040±0.0031 (HN) to 0.0102±0.0053 (HS; Table 2). The mean haplotype diversity of the two foreign cattle breeds was 0.814±0.071, while the mean nucleotide diversity was 0.0040±0.0031. Qinchuan (QC), Nanyang (NY), Zaosheng (ZS), Mongolian (MG), and Xianan (XN) had a positive Tajima's D value, while the other seven cattle breeds had a negative Tajima's D value. All P values of the Tajima's D neutrality test were larger than 0.05 for all cattle breeds, except for Enshi (ES) and Denan (DN).

### 3.3 Phylogenetic analysis

Pairwise genetic distances among the 12 cattle breeds computed using the Kimura two-parameter model are shown in Table 4. Genetic distances ranged from 0.001 to 0.010. The longest genetic distances (0.010) were between Japanese Black (JB) and Enshi (ES), Japanese Black (JB) and Denan (DN), Red Steppe (HN) and Enshi (ES), and Red Steppe (HN) and Denan (DN). The shortest distance (0.001) was between Japanese Black (JB) and Red Steppe (HN).

**Table 4:** Genetic distances among 12 cattle breeds based on mtDNA 16S rRNA gene sequences.

Breed${}^{*}$	JX	XN	ZS	MG	QC	NY	HN	HS	AN	ES	DN	JB
JX	–											
XN	0.006	–										
ZS	0.006	0.005	–									
MG	0.006	0.005	0.005	–								
QC	0.007	0.006	0.005	0.006	–							
NY	0.007	0.006	0.006	0.006	0.006	–						
HN	0.007	0.006	0.004	0.005	0.006	0.008	–					
HS	0.008	0.007	0.006	0.006	0.007	0.008	0.006	–				
AN	0.006	0.005	0.005	0.005	0.006	0.006	0.005	0.006	–			
ES	0.006	0.005	0.006	0.008	0.006	0.004	0.010	0.009	0.006	–		
DN	0.007	0.005	0.006	0.008	0.006	0.004	0.010	0.009	0.006	0.003	–	
JB	0.007	0.007	0.005	0.006	0.006	0.008	0.001	0.005	0.006	0.010	0.010	–

${}^{*}$ Breed abbreviations given in Table 1.

**Figure 1:**
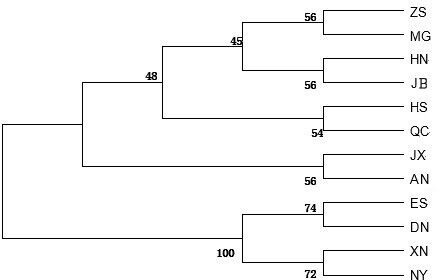
UPGMA dendrogram for 12 cattle breeds based on genetic distances between mtDNA 16S rRNA gene sequences computed using the Kimura two-parameter model. Abbreviations given in Table 1. Japanese Black (*Bos taurus*) and Angus (*Bos taurus*) were control breeds.

A dendrogram for the 12 cattle breeds was constructed with the UPGMA (unweighted pair group method with arithmetic mean) method using the genetic distances computed with the Kimura two-parameter model (Fig. 1). The dendrogram separated cattle breeds into two clusters: Enshi (ES), Denan (DN), Xianan (XN) and Nanyang (NY) belonged to one cluster while the other eight cattle breeds (including the two controls) belonged to another cluster.

**Figure 2:**
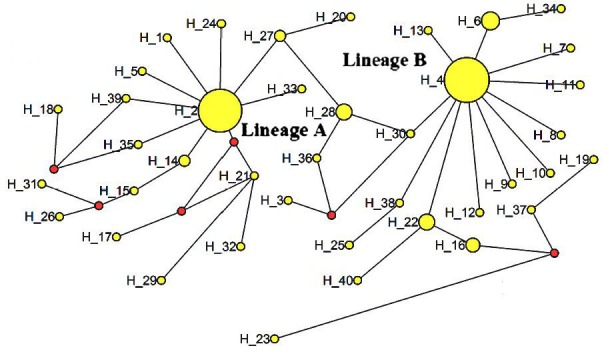
Median-joining network of 12 cattle breeds based on mtDNA 16s rRNA gene sequences (circle areas proportional to sample sizes).

**Figure 3:**
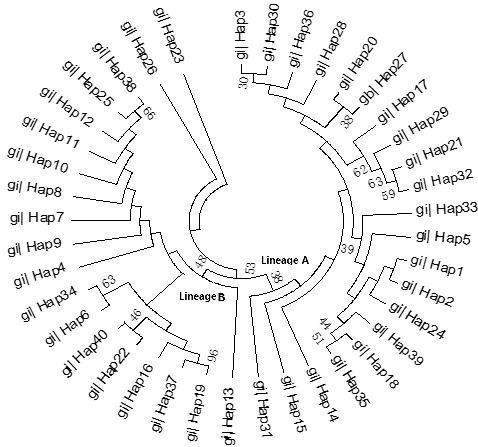
Maximum likelihood tree of 40 haplotypes from 12 cattle breeds based on mtDNA 16S rRNA gene sequences.

The ML tree constructed with the 40 identified haplotypes clearly showed two main lineages: A and B (Fig. 2). A MJ network was also constructed with these 40 haplotypes (Fig. 3). Two seemingly ancestral haplotypes were identified, haplotype H2 in lineage A (shared by eight breeds; Table 3) and haplotype H4 in lineage B (shared by 11 breeds; Table 3). Interestingly, haplotype H23 was located off the center of the network and far from the other haplotypes.

## 4 Discussion

Previous studies reported a base composition bias towards A + T in the mtDNA D-loop region (Lei et al., 2006; Yang et al., 2014) and in the *cyt b* gene (Cai et al., 2007; Kim et al., 2013) in cattle. In fact, earlier research (Perna and Kocher, 1995; Saccone et al., 1999, 2000) indicated that mitochondrial genomes of many organisms including mammals were guanine and cytosine (GC) poor. Sequencing of the mtDNA 16S rRNA gene in animals of the 12 cattle breeds here also proved to have a low GC content. This mitochondrial sequence information would be a valuable resource for bovine phylogenetic analyses. Phylogeneticists discovered that the variation in GC content among organisms could seriously affect reconstructions of evolutionary history because tree-building techniques frequently assigned unrelated species with similar GC content to the same group (Mooers and Holmes, 2000). No significant differences in nucleotide composition of the mtDNA 16S rRNA gene were found among the 12 cattle breeds. The stable GC content was helpful for the utilization of the mtDNA 16S rRNA gene as a cattle phylogenetic analysis marker.

The detected 78 polymorphic sites indicated a high mutation rate in the mtDNA 16S rRNA gene of the 251 sampled animals. The frequency of transitions in the mtDNA D-loop sequence has been higher than that of transversions throughout the bovine evolutionary process (Lai et al., 2006; Liu et al., 2006). Similarly, the percentage of transitions in the mtDNA 16S rRNA gene of the 12 breeds here was 24.6 % higher than that of transversions. This finding agrees with the regularity of mtDNA evolution in mammals (Chen et al., 1993; Loftus et al., 1994; Yang et al., 2014).

Lower average haplotype diversity (h=0.903±0.077) and higher average nucleotide diversity (π=0.0071±0.0039) in the mtDNA 16S rRNA gene here were found than for values in mtDNA D-loop sequences reported for Chinese Wuchuan Black cattle (h=0.909 and π=0.055) (Yang et al., 2014), 16 native Chinese cattle breeds of *Bos taurus* and *Bos indicus* origin (h=0.932 and π=0.023) (Zhang et al., 2009), and 14 Chinese cattle populations (h=0.904±0.008 and π=0.0257±0.0001) (Xia et al., 2019). Interestingly, the *cyt b* gene in Chinese cattle breeds showed lower haplotype diversity (h=0.848) and higher nucleotide diversity (π=0.00923) (Cai et al., 2007) than the corresponding values for the mtDNA 16S rRNA gene. This indicated the existence of different diversities among different mtDNA genes in Chinese cattle breeds. In addition, the two foreign cattle breeds had lower average haplotype diversity (0.814±0.071) and nucleotide diversity (0.0040±0.0031) compared with the 10 Chinese cattle breeds, which indicated the abundant diversities of these Chinese breeds.

Animal breeds had significantly positive values for Tajima's D neutrality statistic, indicating a decrease in population size or selection favoring heterozygotes in multiple loci. Some native Chinese breeds became extinct before the necessary conservation efforts could be carried out: Gaotai cattle and Yangba cattle in Gansu Province, for example, which went extinct more than 30 years ago (Feng et al., 1997; Ma et al., 2002). QC, NY, ZS, and MG are well-known native Chinese cattle breeds and XN is a new Chinese crossbreed produced by crossbreeding Charolais with native Nanyang cattle. Although the values were not significant (P>0.10), positive Tajima's D values were detected in these five breeds. Thus, if population sizes of the QC, NY, ZS, and MG breeds are decreasing, this trend may need to be reversed if these breeds are to be preserved. The other seven cattle breeds (JX, ES, HS, HN, DN, AN, and JB) had negative Tajima's D values, especially the ES and DN breeds (0.01<P<0.05). This indicated that these breeds are increasing their numbers as well as undergoing selection of superior animals for traits of economic importance as well as culling of less desirable animals. With the support of a famous Chinese project named “National Beef Cattle Industrial Technology System”, the population size of some native Chinese breeds, such as JX and ES, is increasing. Further, the population size of DN cattle (Gelbvieh × Nanyang crossbreed) is also expanding rapidly with the strong support of the local government.

Uniparental markers such as those in the Y chromosome and mtDNA have been widely used to trace the origin and to conduct phylogenic and diversity analyses in cattle. Short tandem repeats on the Y chromosome (Y-STR) in Chinese Qinchuan cattle revealed that this breed originated primarily from *Bos taurus* (Xin et al., 2010). Paternal origin analysis of Chinese cattle using single-nucleotide polymorphisms on the Y chromosome (Y-SNPs) and Y-STRs showed that Chinese cattle breeds had two distinct paternal origins: northern Chinese cattle were of the taurine group, whereas southern Chinese cattle belonged to the indicine group (Li et al., 2013). Further, recent research with Y-chromosome haplotypes and autosomal variants showed that East Asian cattle populations were mainly composed of three distinct ancestries: an earlier East Asian taurine ancestry, Eurasian taurine ancestry, and a novel Chinese indicine ancestry (Chen et al., 2018). Studies based on the bovine mtDNA D-loop region (Lai et al., 2006; Lei et al., 2006; Yang et al., 2014; Xia et al., 2019) and the *cyt b* gene (Cai et al., 2007) confirmed that Chinese cattle had both taurine and indicine ancestries. These studies also showed that cattle breeds in southern China had higher levels of *Bos indicus* ancestry, whereas breeds in northern China had higher levels of *Bos taurus* ancestry. The 12 cattle breeds in our research were divided into two lineages (Fig. 1). The first lineage contained six Chinese breeds (ZS, MG, HN, HS, QC, and JX) as well as two taurine control breeds (AN and JB). Among the six Chinese breeds, MG is a northern breed while ZS, QC, and JX are located geographically close to the northern region of China. HN is a crossbreed of Shorthorn and Mongolian cattle ancestry, and HS is also a crossbreed of Holstein and native Chinese yellow cattle ancestry. The geographical location and ancestry of these six cattle breeds indicate that they likely have higher levels of *Bos taurus* ancestry than cattle breeds from southern China. The second lineage contained four breeds (ES, DN, XN, and NY) with likely higher percentages of *Bos indicus* ancestry than the six breeds in the first lineage. ES is a southern breed while NY is located geographically close to the southern region of China. DN and XN are crossbreeds with NY as the female parent. Thus, these four Chinese breeds likely have higher levels of *Bos indicus* ancestry than breeds in northern China. In general, results of the phylogenic analysis here were consistent with those of former studies.

The advantage conferred by the two seemingly ancestral haplotypes (Figs. 2 and 3) to the eight breeds in lineage A (haplotype H2) and the 11 breeds in lineage B (haplotype H4) is unclear. Further research on the mtDNA 16S rRNA gene in these and other cattle breeds may shed light on its role concerning adaptability and productivity of cattle in China.

## 5 Conclusions

We investigated the diversity and phylogenic relationships of 12 cattle breeds (10 Chinese breeds and two foreign taurine breeds as controls) using the mtDNA 16S rRNA gene for the first time in China. The base percentages of this gene had a strong bias towards A + T. There were no indels in the gene; only transitions or transversions were detected. The characterization of the gene in these Chinese cattle breeds agreed with the regularity of mtDNA evolution in mammals. The phylogenetic analysis with gene mtDNA 16S rRNA indicated the existence of *Bos taurus* and *Bos indicus* ancestry in Chinese cattle, in agreement with results from analyses with the D-loop and *cyt b* genes. Thus, the mtDNA 16S rRNA gene was a suitable marker for cattle phylogenetic analyses of Chinese cattle breeds.

## Data Availability

The data sets are available from the corresponding author upon request.
